# Use of Contrast-Enhanced Ultrasound to Study Relationship between Serum Uric Acid and Renal Microvascular Perfusion in Diabetic Kidney Disease

**DOI:** 10.1155/2015/732317

**Published:** 2015-05-26

**Authors:** Ling Wang, Jia-Fen Cheng, Li-Ping Sun, Ya-Xiang Song, Le-Hang Guo, Jun-Mei Xu, Tian-Fu Wu, Chandra Mohan, Ai Peng, Hui-Xiong Xu, Xin-Ying Liu

**Affiliations:** ^1^Department of Nephrology and Rheumatology, Shanghai Tenth People's Hospital, Tongji University, No. 301 Yanchangzhong Road, Zhabei District, Shanghai 200072, China; ^2^Department of Ultrasound in Medicine, Shanghai Tenth People's Hospital, Tongji University, No. 301 Yanchangzhong Road, Zhabei District, Shanghai 200072, China; ^3^Department of Biomedical Engineering, University of Houston, 3605 Cullen Boulevard, Houston, TX 77204, USA

## Abstract

*Purpose.* To investigate the relationship between uric acid and renal microvascular perfusion in diabetic kidney disease (DKD) using contrast-enhanced ultrasound (CEUS) method.* Materials and Methods.* 79 DKD patients and 26 healthy volunteers were enrolled. Renal function and urine protein markers were tested. DKD patients were subdivided into two groups including a normal serum uric acid (SUA) group and a high SUA group. Contrast-enhanced ultrasound (CEUS) was performed, and low acoustic power contrast-specific imaging was used for quantitative analysis.* Results.* Normal controls (NCs) had the highest levels of AUC, AUC1, and AUC2. Compared to the normal SUA DKD group, high SUA DKD patients had significantly higher IMAX, AUC, and AUC1 (*P* < 0.05). DKD patients with low urinary uric acid (UUA) excretion had significantly higher AUC2 compared to DKD patients with normal UUA (*P* < 0.05).* Conclusion.* Hyperuricemia in DKD patients was associated with a renal ultrasound image suggestive of microvascular hyperperfusion. The CEUS parameter AUC1 holds promise as an indicator for renal microvascular hyperperfusion, while AUC2 might be a useful indicator of declining glomerular filtration rate in DKD patients with decreased excretion of uric acid.

## 1. Introduction

Diabetic kidney disease (DKD) remains one of the most common causes of chronic kidney disease (CKD) and end stage of renal disease (ESRD), often associated with higher prevalence of cardiovascular events and higher mortality and morbidity [1, 2].

Recently, cumulative investigations have demonstrated that serum uric acid is an important factor for progression of DKD [3, 4]. During a 5-year follow-up period, Zoppini et al. found that hyperuricemia was an independent risk factor for the development of incident CKD in type 2 diabetic patients [5]. It was also reported that lowering serum uric acid could prevent early renal function loss in diabetes [6]. Moreover, hyperuricemia is related to an increased risk of macrovascular disease [7], coronary heart disease [8], and atrial fibrillation [9] in DKD patients. Hyperuricemia may cause endothelial dysfunction in CKD and diabetic patients [10, 11]. All these data indicated that serum uric acid was involved in endothelial dysfunction and renal vascular disease. Hence, it is conceivable that serum uric acid may be associated with the regulation of renal perfusion, which warrants further investigation.

Unfortunately, there is a lack of tools for the direct monitoring of real-time changes of renal microvascular perfusion in humans. Although, magnetic resonance imaging (MRI) could be used for renal perfusion evaluation [12, 13], its application was limited because of the nephrogenic toxicity of its contrast. Consequently, most of the experiments about the relationship between uric acid and vascular function in humans are based on in vitro studies or studies of nonrenal macrovessels. Contrast-enhanced ultrasound (CEUS) uses contrast agents consisting of tiny gas-filled microbubbles and the size of red blood cells [14]. The microbubbles which could be considered as red blood cell tracer agents would diffuse into the blood, get metabolized by the liver, and exhale through the lungs [15]. Moreover, these agents could not be filtered or secreted by the kidney, and there is no risk of nephrotoxicity [16]. Thus, CEUS has been applied to diverse renal evaluations, such as acute pyelonephritis, renal tumors, cystic lesions, vascular insults, and renal transplantation [16, 17]. Our previous study had reported that CEUS parameters such as AUC could be used for the diagnosis of the renal microvascular damage in early and late stages of DKD [18]. Moreover, we also validated the above findings in an animal model [19]: we found that CEUS was useful for the dynamic assessment of renal perfusion and it was associated with changes in renal pathology. We propose applying the CEUS technique to monitor the effects of uric acid on renal microvascular perfusion in DKD.

## 2. Materials and Methods

### 2.1. Study Design and Patients Involved

79 DKD patients were recruited from the Department of Nephrology and Rheumatology, Shanghai Tenth People's Hospital, Tongji University (China). The inclusion criteria were DKD patients with estimated glomerular filtration rate (eGFR) > 30 mL/min/1.73 m^2^. eGFR was calculated based on the modified-MDRD equation [20]. DKD was confirmed according to US Kidney Disease Outcome Quality Initiatives (K/DOQI) guidelines (https://www.kidney.org/professionals/KDOQI/guidelines_commentaries). 26 healthy volunteers were enrolled as normal controls (NCs). The healthy adults had no history of diabetes and kidney disease and had normal blood glucose and serum creatinine level. Exclusion criteria included non-DKD patients, eGFR ≤ 30 mL/min/1.73 m^2^, egg allergy, severe heart, brain or pulmonary disease, pregnancy, and those of age >80 year old. The recruitment of all subjects was in accordance with institutional review board-approved guidelines. Before examination of CEUS, all subjects had blood drawn for blood urea nitrogen (BUN), serum creatinine (SCr), and serum uric acid (SUA) tests and had urine collection for detections of urine transferrin (TRF), *α*1-microglobulin, *α*1-microglobulin/creatinine ratio (*α*1-MG/UCR), microalbumin (MALB), microalbumin/creatinine ratio (MALB/UCR), and retinol binding protein (RBP). 24 h urine collections were used for urinary urine acid and protein tests. Serum uric acid (SUA) levels ≥360 *μ*mol/L (6 mg/dL) in females and ≥420 *μ*mol/L (7 mg/dL) in males were considered as high SUA; otherwise, the levels were considered as normal SUA. Thus, DKD patients were subdivided into 2 subgroups: normal SUA group (*n* = 44) and high SUA group (*n* = 35). The characteristics of the study groups are shown in Table 1.

### 2.2. CEUS

All imaging was performed in individuals whose blood pressure was controlled within the range of 100–140/60–90 mmHg. The ultrasonographic device, GE Logiq E9, was used, as follows. Both kidneys were scanned to capture the kidney's position, form, echo, and size (length and width). In order to ensure the quality of display, the probe frequency, gain, focus point, and scope were adjusted over the area of kidney cortex and also oriented at the coronal section of the kidneys. Each subject was instructed to breathe normally. Doppler was applied for renal blood flow test. SonoVue (Bracco, Italy) was prepared according to the manufacturer's instructions. After the contrast-specific imaging mode was initiated, 1.2 mL of the contrast agent was administrated through the antecubital vein using a 20-gauge intravenous cannula (Venflon; Becton Dickinson, Helsingborg, Sweden), followed by a 5 mL flush of 0.9% sodium chloride solution. While the contrast agent was being injected, the largest section of the kidney was selected, and echo changes from the kidneys were continuously captured over 5 minutes. This is referred to as a real-time low acoustic power contrast-specific imaging.

### 2.3. Image Analysis

**Figure 1:**
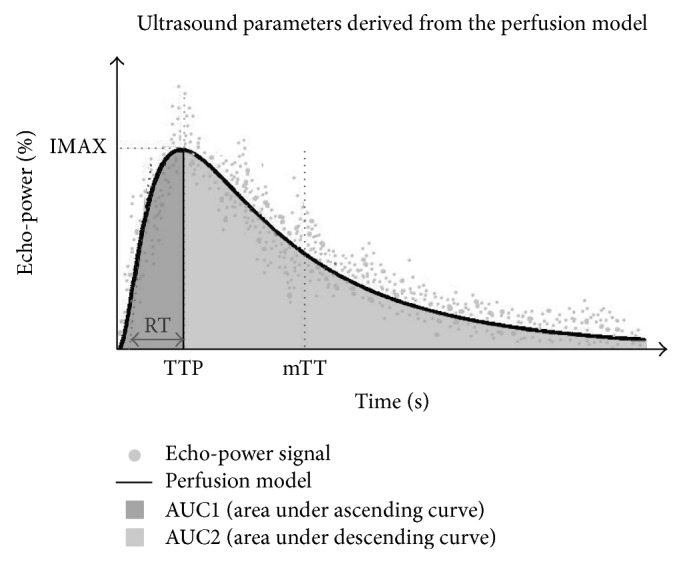
The parameters derived from the CEUS perfusion model. These include maximum intensity (IMAX, with respect to IMAX of the reference ROI), rise time (RT, independent of the time origin), time to peak (TTP), and mean transit time (mTT, corresponding to the center of gravity of the perfusion model). AUC (area under curve) is divided into two parts including AUC1 (area under ascending curve) and AUC2 (area under descending curve).

SonoLiver software 1.1 from TomTec Imaging Systems GmbH (Germany) was used for image analysis. The analysis procedure is performed following the instruction of the manufacturer. Simply, two regions of interest (ROI) were defined, a reference ROI and an analysis ROI. The reference ROI region was set at the 10-clock position in each kidney image, and the analysis ROI region was set at the 12-clock position in each image. The echo-power signals at different times of perfusion were analyzed by the software and summated to generate a perfusion model. The parameters derived from the perfusion model are showed in Figure 1, including maximum intensity (IMAX, with respect to the IMAX of the reference ROI), rise time (RT, independent of the time of origin), time to peak (TTP), and mean transit time (mTT, corresponding to the center of gravity of the perfusion model). Then, the area under the perfusion curve (AUC) to infinite time was calculated. AUC1 was defined as area under the ascending curve, and AUC2 was defined as area under the descending curve. To increase the accuracy, each analysis was repeated two times. The final reported results of IMAX, RT, TTP, mTT, and AUC represent the average value of each parameter captured from both kidneys of each individual.

### 2.4. Statistical Analysis

Data with normal distribution were expressed as mean ± SD, or as median (25%–75% interquartile) if skewed distribution. Comparisons across the three groups and between two groups were performed using the Kruskal-Wallis test followed by the Mann-Whitney *U* test for data with skewed distributions, or one way analysis of variance (ANOVA) followed by LSD test for normally distributed data. Differences in gender distribution and hypertension incidence among groups were analyzed using the chi-square test. SPSS 18.0 (Chicago, IL, USA) was used for statistical calculations. A value of *P* < 0.05 was considered statistically significant. GraphPad Prism 5.0 was used for area under curve (AUC) analysis and graphics.

## 3. Results

### 3.1. Clinic Information about the Study Groups

As shown in Table 1, there was no difference in the distribution of age, gender, and body mass index (BMI) among all the groups. Comparing to the normal controls (NCs), DKD patients had decreased eGFR (*P* < 0.05) and increased levels (*P* < 0.05) of blood urine nitrogen (BUN), serum creatinine (SCr), and serum uric acid (SUA). Also, NCs had lower urine protein markers than DKD patients (*P* < 0.05), including transferrin (TRF), microalbumin (MALB), retinol binding protein (RBP), *α*1-microglobulin (*α*1-MG), and 24 h urine protein. Compared to normal SUA group, DKD patients with high SUA exhibited significantly increased BUN, SCr, and SUA (*P* < 0.05) and decreased urinary uric acid (UUA) and eGFR (*P* < 0.05) but similar excretion of urine protein markers and hypertension incidence.

### 3.2. High Serum Uric Acid with Enhanced Renal Perfusion in DKD

**Figure 2:**
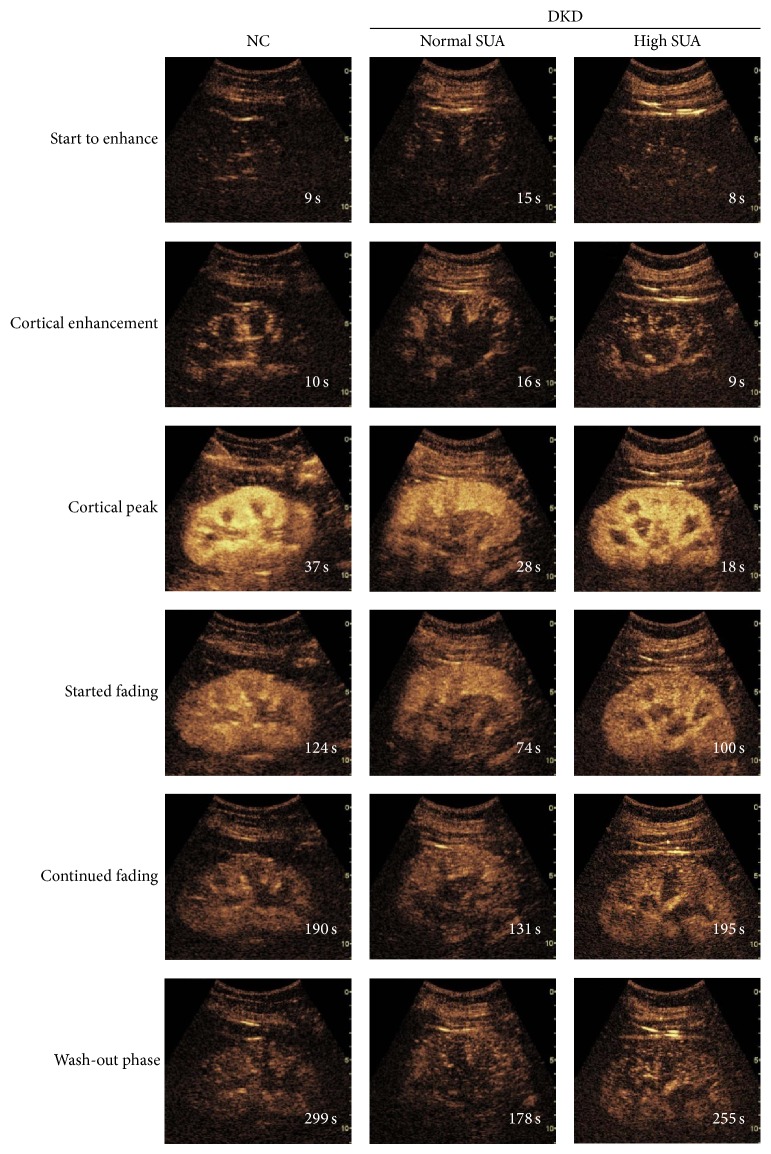
Representative serial contrast-enhancement images in groups. NC, normal control; SUA, serum uric acid; DKD, diabetic kidney disease. All the subjects went through 6 stages including “start to enhance,” “cortical enhancement,” “cortical peak,” “started fading,” “continued fading,” and “wash-out phase.” Individuals in normal SUA and high SUA DKD groups reached their “cortical peak” stage faster than NC, and also they took less time to progress into the “wash-out phase” than the NC group.

**Figure 3:**
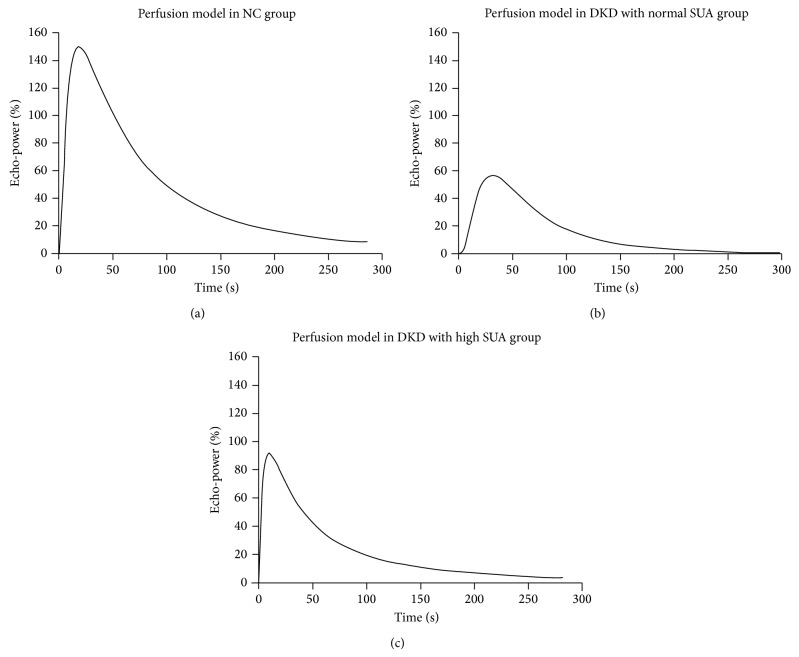
Representative CEUS perfusion curves according to the echo-power signal over the time-course of perfusion. NC, normal control; SUA, normal serum uric acid; DKD, diabetic kidney disease. Each curve has an asymmetrical single-peak curve with an obvious ascending slope, peak, and descending slope. NC had the largest area under curve (AUC) among the three groups. The high SUA DKD group had higher IMAX and larger area under curve than the normal SUA DKD group.

Representative serial contrast-enhancement images captured from the NC, normal SUA, and high SUA DKD groups are shown in Figure 2. All subjects were imaged through 6 stages including “start to enhance,” “cortical enhancement,” “cortical peak,” “started fading,” “continued fading,” and “wash-out phase.” Individuals in normal SUA and high SUA groups reached their “cortical peak” stages faster than NC, and also they took less time to progress into “wash-out” phase than the NC group, especially in normal SUA group (Figure 2). The corresponding curves according to the echo-power signal over the time-course of perfusion are exhibited in Figure 3. Each curve has an asymmetrical single-peak curve with an obvious ascending slope, peak, and descending slope. NC had the largest area under curve (AUC) among the three groups. The high SUA DKD group had higher IMAX and larger area under curve than normal SUA DKD group. Quantitative analysis of the CEUS parameters (Table 2) showed that, when compared to the DKD patients, NC had significant higher levels (*P* < 0.05) of AUC, AUC1, and AUC2. The high SUA DKD group had significantly higher levels (*P* < 0.05) of IMAX, AUC, and AUC1 compared to the normal SUA DKD group, but there was no significant difference in AUC2 between these two groups. Other parameters such as RT, TTP, and mTT were similar among the three groups.

Since eGFR in the high SUA DKD group was significantly decreased compared to that in the normal SUA DKD group (median 54.7 versus 81.79 mL/min/1.73 m^2^, Table 1), we further divided DKD patients into two subgroups based on eGFR level including patients with eGFR ≥ 60 and patients with eGFR < 60 mL/min/1.73 m^2^ (Table 3). The clinical characteristics of the subjects with different eGFR are listed in Supplemental Table 1 available online at http://dx.doi.org/10.1155/2014/732317: DKD patients with lower eGFR (<60 mL/min/1.73 m^2^) had higher levels of urine proteins, BUN, and SUA than higher eGFR patients (≥60 mL/min/1.73 m^2^, *P* < 0.05). As shown in Table 3, NC had higher level of AUC and AUC1 than the other groups (*P* < 0.05), but there were no differences in all the CEUS parameters between DKD patients with eGFR ≥ 60 mL/min/1.73 m^2^ and patients with eGFR < 60 mL/min/1.73 m^2^. These findings indicated that eGFR did not have a major impact on the CEUS imaging results within the DKD patients in this study.

### 3.3. Low Urinary Uric Acid with Decreased Clearance of Renal Perfusion in DKD

**Figure 4:**
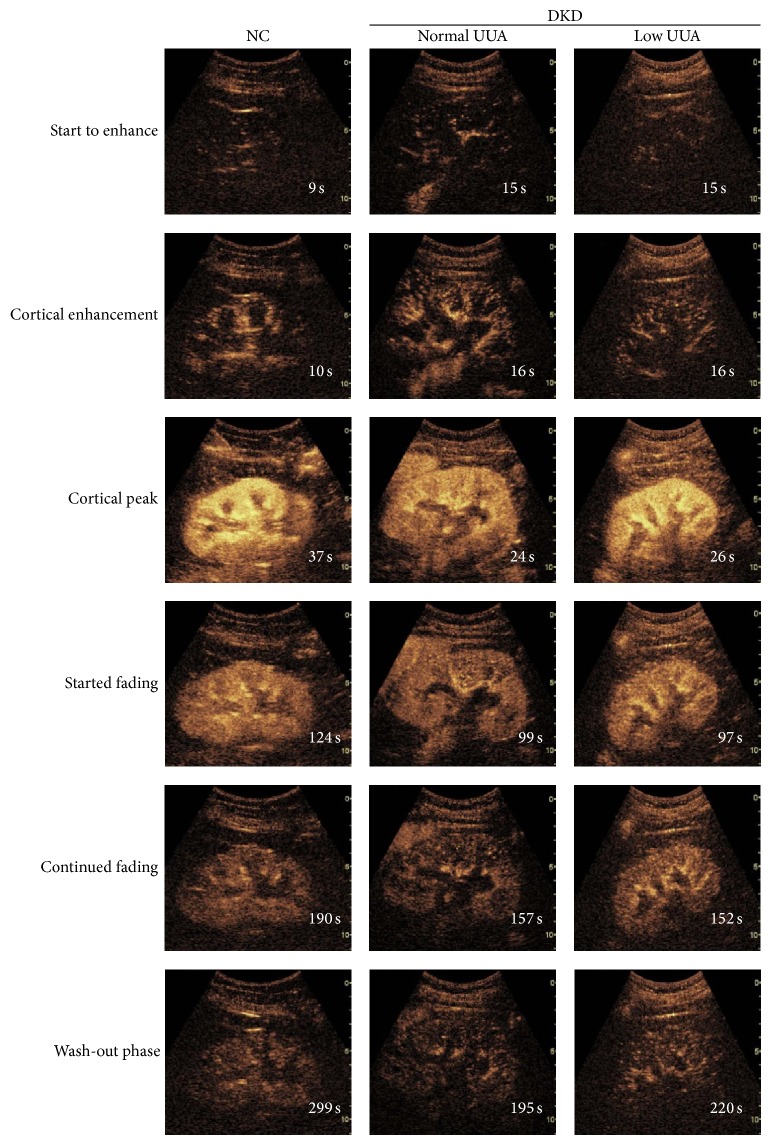
Representative serial contrast-enhancement images in groups. NC, normal control; UUA, urinary uric acid; DKD, diabetic kidney disease. All the subjects went through 6 stages including “start to enhance,” “cortical enhancement,” “cortical peak,” “started fading,” “continued fading,” and “wash-out phase.” The image of cortical peak in low UUA DKD group was brighter than normal UUA DKD, and low UUA DKD group reached the “wash-out phase” slower than the normal UUA DKD group but faster than the NC group.

**Figure 5:**
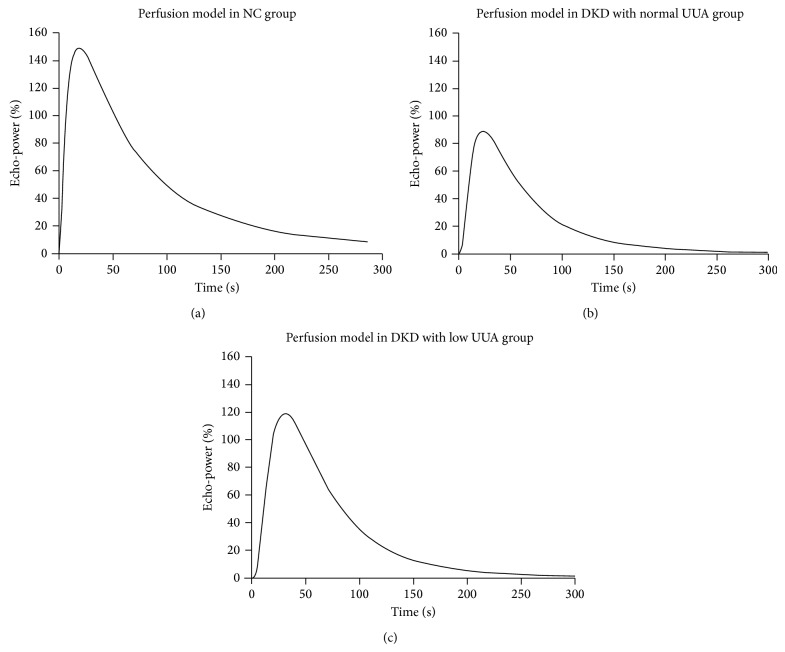
Representative CEUS perfusion curves corresponding to the echo-power signal over the time-course of perfusion. NC, normal control; UUA, normal urinary uric acid; DKD, diabetic kidney disease. The curves showed that NC group exhibited the largest curve among the three groups, and low UUA DKD group had a larger perfusion curve than the normal UUA DKD group.

In our study, NC had similar level of UUA as normal SUA, but DKD patients in high SUA group had significantly lower levels of UUA than the other two groups (*P* < 0.05, Table 1). Thus, DKD patients were further classified into normal UUA and low UUA group and reanalyzed. The clinical characteristics of these two groups with different excretion of UUA are listed in Supplemental Table 2. Representative serial contrast-enhancement images and curves in NC, normal UUA, and low UUA DKD groups are shown in Figures 4 and 5. Quantitative analysis indicated that DKD patients with low UUA had significantly higher level of AUC, especially AUC2, than patients with normal UUA (*P* < 0.05, Table 4). As before, AUC, AUC1, and AUC2 were increased significantly in NC compared to the other groups (*P* < 0.05, Table 4). However, there was no difference among DKD groups in IMAX, RT, TTP, and mTT (Table 4). Moreover, eGFR in the low UUA group (69.98 ± 7.06) trended to be lower than in normal UUA group (82.29 ± 6.08), but the difference did not reach statistical significance (*P* > 0.05). The larger AUC, especially AUC2 in the low UUA DKD patients, together with their lower eGFR, may represent stronger renal perfusion with decreased clearance.

## 4. Discussion

CEUS is a powerful imaging technology for the evaluation of renal perfusion with safety and effectiveness [16, 18]. Due to differences in blood volume of each kidney, different amounts of contrast microbubbles permeate into the renal tissue, correlating with different intensities of contrast-enhancement signal [21]. In our study, all DKD patients had eGFR > 30 mL/min/1.73 m^2^, which meant they had early to moderate stage of kidney disease. By quantitative analysis, we found that AUC, AUC1, and AUC2 are decreased significantly in DKD patients (*P* < 0.05, Table 2). This means that the renal perfusion and clearance ability in DKD patients was impaired compared to NC. Typical pathological changes of diabetic nephropathy include proliferation of extracellular matrix and destroyed renal construction which could lead to declined renal filtration rate in developed disease. According to the results, decreased AUC1 and AUC2 could reflect the decline of renal filtration rate in early to moderated stage of DKD. These findings confirm our previous study [18] indicating that AUC could help to assess and diagnose renal microvascular damage in DKD and also in resonance with Staub et al.'s work [22].

It is interesting from this study that DKD patients with high serum uric acid (SUA) had significantly higher levels of IMAX, AUC, and especially AUC1 than normal SUA DKD patients (Table 2). Moreover, when we looked at the clinical data (Table 1), high SUA DKD patients exhibited similar levels of various urine protein markers (such as TRF, MALB, RBP, and *α*1-MG) but significantly decreased eGFR and urinary uric acid (UUA) compared to normal SUA patients (*P* < 0.05, Table 1). Urinary TRF and MALB represent glomerular injuries [23, 24] and urine RBP and *α*1-MG represent renal tubular damages [25, 26]. Thus, individuals in our DKD groups had obvious kidney injuries. Since IMAX represented the perfusion intensity and AUC1 reflects the volume of renal perfusion, it is reasonable to posit that high SUA may be associated with renal hyperperfusion and decreased GFR in the early to moderate stage of DKD. Previous research has established the connection between hyperuricemia and DKD. A cohort study had shown that serum uric acid (SUA) level is an independent risk factor for renal dysfunction [27], especially in DKD [28]. A study of the natural history of DKD also supported that SUA together with vascular comorbidities strongly indicated faster progression of DKD [29]. The potential mechanisms responsible could be endothelial dysfunction caused by hyperuricemia [30, 31]. Our study not only confirmed the association between high SUA and decreased renal function of DKD, but also suggested that renal microvascular hyperperfusion may be a feature of early to moderate renal dysfunction in DKD.

Our study also showed that DKD patients with low UUA had significantly higher AUC, especially AUC2 when compared to normal UUA DKD patients (*P* < 0.05, Table 4). The clinical data (Supplemental Table 2) revealed that low UUA patients seemed to have a trend of declined eGFR compared to normal UUA patient (median 64.38 versus 75.33 mL/min/1.73 m^2^), though not statistically different (*P* > 0.05, Supplemental Table 2). Since AUC2 represented the clearance of renal perfusion, these results may suggest decreased clearance of perfusion in DKD patients with low UUA. Whether AUC2 could be a more sensitive indicator than eGFR in tracking the decline of renal function warrants systemic study. Moreover, our results revealed that low UUA patients had higher excretion rate of urinary microalbumin (*P* < 0.05) compared to normal UUA patients. Since microalbuminuria is a marker of endothelial dysfunction [32, 33], low UUA DKD patients may have underlying the glomerular endothelial dysfunction. Thus, taking the data together, high SUA was related to CEUS AUC1, but low UUA was related to CEUS AUC2 in DKD patients.

Our previous study [18] reported that AUC could distinguish DKD patients with CKD 1 (eGFR > 90 mL/min/1.73 m^2^) and 2 stages (eGFR 60–90 mL/min/1.73 m^2^) from CKD 4 (eGFR 15–30 mL/min/1.73 m^2^) and 5 stages (eGFR < 15 mL/min/1.73 m^2^). Here, the present study failed to distinguish DKD patients with different eGFR levels, which was different from our previous study. It is possible that the present study included intermediate CKD stage 3 patients (eGFR 30–60 mL/min/1.73 m^2^) are quite different from the previous study. Thus, it is possible that CEUS may not be sensitive enough in distinguishing CKD stage 3 patients from CKD stage 1 to 2 patients with DKD.

In summary, uric acid may be associated with the development of renal microvascular hyperperfusion in DKD. The CEUS parameter AUC1 holds promise as an indicator for renal microvascular hyperperfusion, while AUC2 may hold promise as a predictor of declined glomerular filtration rate in patients with decreased excretion of urinary uric acid. However, these predictions warrant further validation. CEUS provides us a real-time and dynamic platform for investigating the effects of hyperuricemia on renal vascular perfusion.

## Supplementary Material

The clinical characteristics of groups with different eGFR and urinary uric acid were revealed in Supplemental Tables.

## Supplementary Material

The clinical characteristics of groups with different eGFR and urinary uric acid were revealed in Supplemental Tables. Supplemental table 1 showed that DKD patients with lower eGFR (<60 mL/min/1.73m2) had higher levels of urine proteins, BUN, and SUA than higher eGFR patients (≥60 mL/min/1.73m2, p <  0.05). Supplemental table 2 showed that low UUA patients had similar levels of urine proteins, but had a trend of declined eGFR compared to normal UUA patient, though not statistically different (p > 0.05).

## Figures and Tables

**Table 1:** Characteristics with study groups^§^.

Parameters	Normal control (NC)	Diabetic kidney damage (DKD)	
		Normal SUA^▽^	High SUA^▽^
Number	26	44	35
Age (year)	57.1 ± 6.4	59.9 ± 8.3	61.0 ± 10.4
Female (%)	50.0 (13/26)	52.3 (23/44)	65.7 (23/35)
BMI^1^ (kg/m^2^)	24.8 ± 5.3	26.0 ± 4.2	26.1 ± 5.1
Hypertension (%)	0^*^	81.8 (36/44)^∆^	88.6 (31/35)^∆^
eGFR^2^ (mL/min/1.73 m^2^)	127.9 (108.1–139.1)^*^	81.79 (55.7–118.7)^∆^	54.70 (38.7–85.9)^∗∆^
CKD stage^3^		2.0 (1–3)	3 (2-3)^*^
BUN^4^ (mmol/L)	5.20 (4.6–5.7)^*^	6.9 (5.7–8.3)^∆^	8.2 (6.6–11.2)^∗∆^
SCr^5^ (*μ*mol/L)	57.95 (52.5–65.7)^*^	85.6 (58.4–115.9)^∆^	119.3 (78.4–160.7)^∗∆^
SUA (*μ*mol/L)	255.19 ± 68.25^*^	314.5 ± 58.9^∆^	470.6 ± 80.1^∗∆^
UUA^6^ (mmol/24 h)	3.1 ± 1.0	3.0 ± 1.3	2.5 ± 1.1^∗∆^
Urine TRF^7^ (mg/L)	0.0 (0-0)^*^	9.0 (1.1–82.5)^∆^	18 (1.4–64.0)^∆^
*α*1-MG/UCR^8^ (g/mol)	0.0 (0-0)^*^	3.2 (80–11.31)^∆^	4.05 (0–8.6)^∆^
MALB^9^ (mg/L)	13.75 (12.5–21.4)^*^	265.6 (67.9–495.9)^∆^	335.3 (121.1–576.5)^∆^
MALB/UCR^10^ (g/mol)	2.81 (2.3–4.0)^*^	40.04 (7.3–97.0)^∆^	59.90 (17.5–91.8)^∆^
Urine RBP^11^ (mg/L)	0.60 (0.5-0.6)^*^	2.6 (1.2–3.6)^∆^	2.8 (2.0–3.6)^∆^
Urine protein (g/24 h)	0.055 (0.03–0.09)^*^	0.57 (0.1–2.4)^∆^	1.0 (0.2–2.3)^∆^

^§^Values are represented as mean ± standard error, median (25%–75% interquartile), or percentage where appropriate. ^▽^SUA, serum uric acid. SUA ≥360 *μ*mol/L (6 mg/dL) in females and ≥420 *μ*mol/L (7 mg/dL) in males are considered as high SUA; otherwise, the levels are considered as normal SUA. ^*^ *P* < 0.05, compared to DKD patients with normal SUA; ^∆^ *P* < 0.05, compared to normal control.

^1^BMI, body mass index; ^2^eGFR, estimated glomerular filtration rate; the calculation is based on the modified-MDRD equation; ^3^CKD stage, chronic kidney disease stages are classified according to K/DOQI CKD guideline (https://www.kidney.org/professionals/KDOQI/guidelines_commentaries); ^4^BUN, blood urea nitrogen; ^5^SCr, serum creatinine; ^6^UUA, urinary uric acid; ^7^TRF, transferrin; ^8^ *α*1-MG/UCR, urinary *α*1-microglobulin/creatinine ratio; ^9^MALB, urinary microalbumin; ^10^MALB/UCR, urinary microalbumin/creatinine ratio; ^11^RBP, retinol binding protein.

**Table 2:** Ultrasound parameters in the different study groups^§^.

Parameters	Normal control (NC)	Diabetic kidney disease (DKD)	
		Normal SUA^▽^	High SUA^▽^
Number	26	36	29
IMAX (%)	104.28 ± 21.63	97.13 ± 19.19	108.50 ± 17.72^*^
RT (s)	20.65 ± 6.17	17.92 ± 5.28	19.58 ± 5.89
TTP (s)	22.27 (15.4–31.0)	19.74 (15.2–26.2)	21.36 (16.6–28.6)
mTT (s)	88.54 ± 30.56	89.30 ± 28.08	91.70 ± 30.82
AUC	8351.81 ± 2153.28^*^	6832.63 ± 1497.06^∆^	7767.41 ± 1762.26^*^
AUC1	1795 (1439–2257)^*^	1148 (981.8–1396)^∆^	1411 (1056–1792)^∗∆^
AUC2	6549.82 ± 1924.37^*^	5646.39 ± 1282.94^∆^	6337.41 ± 1540.97

^§^Values are represented as mean ± standard error. ^▽^SUA, serum uric acid. SUA ≥360 *μ*mol/L (6 mg/dL) in females and ≥420 *μ*mol/L (7 mg/dL) in males are considered as high SUA; otherwise, they are considered as normal SUA. ^*^ *P* < 0.05, compared to DKD patients with normal SUA; ^∆^ *P* < 0.05, compared to normal control. Ultrasound parameters abbreviations used are as follows: IMAX, maximum intensity; RT, rise time; TTP, time to peak; mTT, mean transit time; AUC, the area under the perfusion curve; AUC1, area under the ascending curve; AUC2, area under the descending curve.

**Table 3:** Ultrasound parameters of groups with different levels of eGFR^§^.

Parameters	Normal control (NC)	Diabetic kidney disease (DKD)	
		eGFR *⩾* 60 mL/min/1.73 m^2^	eGFR < 60 mL/min/1.73 m^2^
Number	26	29	36
IMAX (%)	104.28 ± 21.63	99.91 ± 18.92	105.05 ± 19.63
RT (s)	20.65 ± 6.17	19.67 ± 5.22	17.41 ± 5.83^Δ^
TTP (s)	23.89 ± 9.05	19.68 ± 7.17	22.46 ± 6.79^Δ^
mTT (s)	88.54 ± 30.56	88.88 ± 24.69	92.22 ± 34.21
AUC	8351.81 ± 2153.28^*^	7333.06 ± 1505.96^∆^	7146.19 ± 1885.03^∆^
AUC1	1795 (1439–2257)^*^	1355 (1103–1610)^∆^	1100 (988.3–1396)^∆^
AUC2	6549.82 ± 1924.37	5989.63 ± 1267.13	5911.31 ± 1641.76

^§^Values are represented as mean ± standard error for normal distributed data; otherwise, as median (25%–75% interquartile). ^*^ *P* < 0.05, compared to DKD patients with normal SUA; ^∆^ *P* < 0.05, compared to normal control. Ultrasound parameters abbreviations used are as follows: IMAX, maximum intensity; RT, rise time; TTP, time to peak; mTT, mean transit time; AUC, the area under the perfusion curve; AUC1, area under the ascending curve; AUC2, area under the descending curve.

**Table 4:** Ultrasound parameters in groups with different urinary uric acid excretion levels^§^.

Parameters	Normal control (NC)	Diabetic kidney damage (DKD)	
		Normal UUA^▽^	Low UUA^▽^
Number	26	25	39
IMAX (%)	104.28 ± 21.63	100.39 ± 20.55	104.06 ± 16.98
RT (s)	20.65 ± 6.17	17.78 ± 5.40	19.77 ± 5.69
TTP (s)	23.89 ± 9.05	20.30 ± 7.07	22.26 ± 6.82
mTT (s)	88.54 ± 30.56	89.92 ± 30.45	92.02 ± 27.68
AUC	8351.81 ± 2153.28^*^	6880.87 ± 1724.24^∆^	7758.08 ± 1470.40^*^
AUC1	1795 (1439–2257)^*^	1175 (972.1–1416)^∆^	1352 (1062–1653)^∆^
AUC2	6549.82 ± 1924.37^*^	5662.69 ± 1498.92^∆^	6377.13 ± 1256.96^*^

^§^Values are represented as mean ± standard error for normal distributed data; otherwise, as median (25%–75% interquartile). ^▽^UUA, urinary uric acid. UUA <2.4 mmol/24 h is considered as low UUA, and UUA 2.4~5.9 mmol/24 is considered as normal UUA. ^*^ *P* < 0.05, compared to DKD patients with normal UUA; ^∆^ *P* < 0.05, compared to normal control. Ultrasound parameters abbreviations used are as follows: IMAX, maximum intensity; RT, rise time; TTP, time to peak; mTT, mean transit time; AUC, the area under the perfusion curve; AUC1, area under the ascending curve; AUC2, area under the descending curve.
