# Supplementary material for: Use of Contrast-Enhanced Ultrasound to Study Relationship between Serum Uric Acid and Renal Microvascular Perfusion in Diabetic Kidney Disease
Source: Biomed Res Int. 2015 May 26;2015:732317. doi: 10.1155/2015/732317 (PMC4464846; doi:10.1155/2015/732317)
Supplement: Supplementary file 1 — The clinical characteristics of groups with different eGFR and urinary uric acid were revealed in Supplemental Tables. Supplemental table 1 showed that DKD patients with lower eGFR (<60 mL/min/1.73m2) had higher levels of urine proteins, BUN, and SUA than higher eGFR patients (≥60 mL/min/1.73m2, p < 0.05). Supplemental table 2 showed that low UUA patients had similar levels of urine proteins, but had a trend of declined eGFR compared to normal UUA patient, though not statistically different (p > 0.05). [file 732317.f1.doc]

Supplemental table 1. Clinical characteristics in subjects with different levels of eGFR^§^

| Parameters | Normal control (NC) | Diabetic kidney disease (DKD) | |
| --- | --- | --- | --- |
|  |  | eGFR≥60 ml/min/1.73m^2^ | eGFR<60 ml/min/1.73m^2^ |
| Number | 26 | 47 | 32 |
| Age (year) | 57.1±6.4 | 59.7±8.6 | 61.3±10.0 |
| Female (%) | 50.0 (13/26) | 66.0(31/47) | 46.9(15/32) |
| BMI^1^ (kg/m^2^) | 24.8±5.3 | 26.3±5.1 | 25.6±4.0 |
| Hypertension (%) | 0* | 78.7 (37/47) ^△^ | 93.8 (30/32) ^△^ |
| eGFR^2^ (ml/min/1.73m^2^) | 127.9(108.1-139.1)* | 94.62(76.0-119.7)^△^ | 42.00(27.0-50.7)*^△^ |
| CKD stage^3^ |  | 1.0(1-2) | 3(3-4)* |
| BUN^4^ (mmol/L) | 5.20(4.6-5.7)* | 6.5(5.3-7.2)^△^ | 9.6(8.2-15.8)*^△^ |
| SCr^5^ (μmol/L) | 57.95(52.5-65.7)* | 74.3(58.4-86.7)^△^ | 152.3(127.3-214.8)*^△^ |
| SUA (μmol/L) | 255.19±68.25* | 360.6±99.1^△^ | 417.5±103.0*^△^ |
| UUA^6^ (mmol/24h) | 3.1±1.0 | 2.9±1.2 | 2.5±1.2 |
| Urine TRF^7^ (mg/L) | 0.0(0-0)* | 6.0(0-29.0)^△^ | 58(10.3-123.0)*^△^ |
| α1-MG/UCR^8^ (g/mol) | 0.0(0-0)* | 0.49(0-5.3)^△^ | 5.12(2.8-17.2)*^△^ |
| MALB^9^ (mg/L) | 13.75(12.5-21.4)* | 189.2(38.2-499.8)^△^ | 448.2(289.5-585.8)*^△^ |
| MALB/UCR^10^ (g/mol) | 2.81(2.3-4.0)* | 34.05(6.8-88.2)^△^ | 58.56(37.0-102.9)*^△^ |
| Urine RBP^11^ (mg/L) | 0.60(0.5-0.6)* | 2.30(1.0-3.6)^△^ | 3.15(2.3-3.6)^△^ |
| Urine protein (g/24h) | 0.055(0.03-0.09)* | 0.18(0.05-1.10)^△^ | 2.04(0.7-4.1)*^△^ |

^§^Values are represented as mean ± standard error, median (25%-75% interquartile) or percentage where appropriate. ^▽^SUA, serum urine acid. SUA ≥360μmol/L in females and ≥420μmol/L in males are considered as high SUA. *P<0.05, compared to DKD patients with normal SUA; ^△^P<0.05, compared to normal control.

^1^BMI, Body mass index; ^2^eGFR, estimated Glomerular Filtration Rate, the calculation is based on the modified-MDRD equation; ^3^CKD stage, chronic kidney disease stages are classified according to K/DOQI CKD guideline (https://www.kidney.org/professionals/kdoqi/ guidelines_ckd/p4_class_g1.htm); ^4^BUN, blood urea nitrogen; ^5^SCr, serum creatinine; ^6^UUA, urinary urine acid; ^7^TRF, transferrin; ^8^α1-MG/UCR, urinary α1-microglobulin/creatinine ratio; ^9^MALB, urinary microalbumin; ^10^MALB/UCR, urinary microalbumin/creatinine ratio; ^11^RBP, retinol binding protein.

Supplemental table 2.

Clinical characteristics in subjects with different levels of urinary uric acid (UUA)^§^

| Parameters | Normal control (NC) | Diabetic kidney disease (DKD) | |
| --- | --- | --- | --- |
|  |  | Normal UUA | Low UUA |
| Number | 26 | 46 | 32 |
| Age (year) | 57.1±6.4 | 58.6±9.6 | 62.8±8.3 |
| Female (%) | 50.0 (13/26) | 54.3(25/46) | 62.5(20/32) |
| BMI^1^ (kg/m^2^) | 24.8±5.5 | 26.9±4.2 | 24.7±5.1 |
| Hypertension (%) | 0* | 83.0 (39/47) ^△^ | 87.5 (28/32) ^△^ |
| eGFR^2^ (ml/min/1.73m^2^) | 127.9(108.1-139.1)* | 75.33(50.7-101.4)^△^ | 64.38 (34.1-106.5)^△^ |
| CKD stage^3^ |  | 2.0(1-3) | 2(1-3) |
| BUN^4^ (mmol/L) | 5.20(4.6-5.7)* | 7.1(5.9-8.8)^△^ | 7.6(5.8-12.1)^△^ |
| SCr^5^ (μmol/L) | 57.95(52.5-65.7)* | 88.75(67.2-129.7)^△^ | 99.05(66.4-175.2)^△^ |
| SUA (μmol/L) | 255.19±68.25* | 377.68±106.1^△^ | 392.05±103.5^△^ |
| UUA^6^ (mmol/24h) | 3.1(2.3-4.2) | 3.4(2.9-4.0) | 1.5(1.2-2.1)* ^△^ |
| Urine TRF^7^ (mg/L) | 0.0(0-0)* | 9.0(0.8-69.5)^△^ | 26.0(7.3-87.0)^△^ |
| α1-MG/UCR^8^ (g/mol) | 0.0(0-0)* | 16.45(0-58.3)^△^ | 28.0(2.6-56.6)^△^ |
| MALB^9^ (mg/L) | 13.75(12.5-21.4)* | 213.5(58.7-473.3)^△^ | 468.7(213.3-571.0)*^△^ |
| MALB/UCR^10^ (g/mol) | 2.81(2.3-4.0)* | 27.05(7.0-86.3)^△^ | 71.43(53.2-101.7)*^△^ |
| Urine RBP^11^ (mg/L) | 0.60(0.5-0.6)* | 2.35(1.2-3.4)^△^ | 3.3(1.7-3.6)^△^ |
| Urine protein (g/24h) | 0.055(0.03-0.09)* | 0.42(0.09-2.73)^△^ | 0.75(0.3-2.0)^△^ |

^§^Values are represented as mean ± standard error, median (25%-75% interquartile) or percentage where appropriate. UUA <2.4 mmol/24h is considered as low UUA, and UUA 2.4~5.9 mmol/24 is considered as normal UUA. *P<0.05, compared to DKD patients with normal UUA; ^△^P<0.05, compared to normal control.

^1^BMI, Body mass index; ^2^eGFR, estimated Glomerular Filtration Rate, the calculation is based on the modified-MDRD equation; ^3^CKD stage, chronic kidney disease stages are classified according to K/DOQI CKD guideline (https://www.kidney.org/professionals/kdoqi/ guidelines_ckd/p4_class_g1.htm); ^4^BUN, blood urea nitrogen; ^5^SCr, serum creatinine; ^6^UUA, urinary urine acid; ^7^TRF, transferrin; ^8^α1-MG/UCR, urinary α1-microglobulin/creatinine ratio; ^9^MALB, urinary microalbumin; ^10^MALB/UCR, urinary microalbumin/creatinine ratio; ^11^RBP, retinol binding protein.
